# Workplace violence against healthcare workers in the Republic of Moldova during COVID-19 pandemic: a cross-sectional study

**DOI:** 10.3389/fpubh.2025.1560096

**Published:** 2025-06-03

**Authors:** Elena Ciobanu, Dumitru Cheptea, Svetlana Cociu, Patricia Maria Marga, Serghei Cebanu, Diana Dulf, Corinne Peek-Asa

**Affiliations:** ^1^Department of Preventive Medicine, “Nicolae Testemitanu” State University of Medicine and Pharmacy, Chisinau, Moldova; ^2^Department of Public Health, Faculty of Political, Administrative and Communication Sciences, Babeș-Bolyai University, Cluj-Napoca, Romania; ^3^Office of Research Affairs, University of California, San Diego, La Jolla, CA, United States

**Keywords:** workplace violence, COVID-19 pandemic, prevention, healthcare workers, professional risk factor, security and training

## Abstract

**Objectives:**

This study aimed to explore healthcare providers’ experiences with workplace violence cases before and during the COVID-19 pandemic and to identify the prevalence of risk factors.

**Methods:**

A cross-sectional study design was conducted among healthcare professionals from six hospitals in Moldova during the COVID-19 pandemic. The study included 189 medical professionals and clinical support staff.

**Results:**

This study surveyed 189 healthcare professionals, mostly aged 40–49 (31.7%), with physicians (43.9%) and nurses (42.3%) being predominant. Departments most represented were infectious disease (28.3%), emergency (21.4%), and intensive care (16.1%). Violence prevention training was lacking (83.6%). Hallways (38.5%) and poorly visible areas (34.3%) were leading environmental risk factors. Workplace violence affected 43.1% of participants. Physical assault and verbal threats have a strong correlation before and during the pandemic (*r* = 0.654; *r* = 0.714), but changes were not statistically significant. Female staff had lower odds of experiencing serious violence (OR = 0.43, *p* = 0.013). Workers with 11+ years’ experience faced fewer incidents, while those with 2–5 years faced a higher risk.

**Conclusion:**

Workplace violence remained prevalent during the COVID-19 pandemic, with statistically significant variation over time, pointing the need for ongoing prevention efforts.

## Introduction

Workplace violence is a growing global concern, particularly in the healthcare sector. The term “workplace violence” is multifaceted, generally defined as the use of force, physical or psychological, against an individual or group in a work-related context, potentially resulting in harm or even death (1). According to the World Health Organization, between 8 and 38% of healthcare professionals worldwide experience some form of workplace violence, predominantly physical (2). Data from the U. S. Bureau of Labor Statistics report an incidence rate of eight assaults per 10,000 healthcare workers – four times higher than the rate observed across other sectors (3).

This trend has become so alarming that the World Medical Association now considers violence against healthcare professionals a global emergency, threatening the sustainability of health systems and patient outcomes equally (4, 5). Verbal, physical, and non-verbal abuse are the most common forms of violence reported in hospital settings, with verbal abuse affecting over half of physicians (6, 7).

The COVID-19 pandemic exacerbated this issue worldwide, with healthcare workers facing not only increased workloads and emotional exhaustion but also intensified aggression from patients and their families (8–10). Public fear, changes in healthcare delivery, and rising professional stress levels may have contributed to the rise in incidents, while also eroding the societal respect traditionally afforded to healthcare providers (11).

Violence in medical workplaces has been strongly associated with negative outcomes such as professional burnout, emotional distress, diminished patient safety, and even withdrawal from the profession (12). Factors like substance abuse among perpetrators, high stress, and physically demanding work environments further aggravate the situation (5, 13–15).

Despite growing international research on this phenomenon, there is a notable absence of empirical data from the Republic of Moldova. Moldovan healthcare professionals continued to work under high stress, with limited systemic support and exposure to risk factors for violence. Yet, workplace violence in Moldovan medical institutions remains understudied and largely undocumented in peer-reviewed literature.

Given the limited national data and the increasing recognition of workplace violence as a barrier to effective healthcare delivery, this study fills a critical knowledge gap. It is the first systematic, cross-sectional assessment of workplace violence experienced by healthcare professionals in Moldova during the COVID-19 pandemic. By using internationally tested tools and drawing comparisons with global findings, this research provides a much-needed evidence base for understanding the prevalence, forms, and risk factors of violence in Moldovan healthcare settings. This study aims to explore healthcare providers’ experiences with workplace violence cases before and during the COVID-19 pandemic and to identify the prevalence of risk factors.

## Methods

### Study setting

A cross-sectional design study was conducted among healthcare professionals from the six major hospitals designated as COVID-19 treatment sites during the pandemic in Chisinau, the Republic of Moldova. A total of 30 hospitals were assigned as COVID-19 treatment sites during the pandemic, and 6 of the largest hospitals were selected. The most advanced specialized hospitals for the treatment of COVID-19 clinical cases were located in the republic’s capital, Chisinau. Accordingly, we selected one regional hospital and five Chisinau hospitals. This sample of six large hospitals represented the highest proportions of COVID-19 patients and served diverse populations, including urban residents, vulnerable groups, and populations with underlying health conditions that made them more susceptible to severe COVID-19 symptoms. The selected hospitals often had existing infrastructure to handle infectious diseases, making them better equipped to manage COVID-19 patients effectively. The hospitals included in the study were: Institute of Emergency Medicine, Clinical Hospital for Infectious Diseases “Toma Ciorbă,” Municipal Clinical Hospital for Children’s Contagious Diseases, “Valentin Ignatenco” Municipal Clinical Hospital, Municipal Clinical Hospital “Sfânta Treime,” Phthisiopneumology Institute “Chiril Draganiuc.”

### Study population

Eligible participants for the study were healthcare workers who provided daily care in wards where COVID-19 patients were treated. The target groups for interviews included doctors, nurses, residents, and clinical support staff. Participation was open to all eligible healthcare providers, regardless of gender, age, seniority, or other individual factors. Within the six hospitals, eligible personnel were invited to participate via email. No formal sample size calculation was performed, as this study had an exploratory and descriptive purpose. A total of 189 healthcare providers expressed interest by signing an informed consent form, forming the study sample, which corresponds to approximately 36% of the estimated 525 eligible staff across the six hospitals. An 18% non-response rate reflects individuals who received the invitation but declined to participate or did not complete the survey.

### Data collection and research tools

The development of a questionnaire for this study was derived from the “Workplace violence in the health sector – Country case studies research instruments – Survey questionnaires” (English version) as set out by an International Labour Office, International Council of Nurses, World Health Organization, Public Services International joint project. The questionnaire was adapted to the particularities of four countries (Armenia, Georgia, the Republic of Moldova and Romania) and the principals’ investigators of the international project iCREATE: Increasing Capacity for Research in Eastern Europe adjusted it based on the learnings from previous common work done. The questionnaire was translated into three languages. Afterward, it was evaluated by experts for clarity and cultural appropriateness through piloting after forward-backward translation.

Healthcare professionals were surveyed both offline (on paper) and online. The questionnaire was sent to the respondents via email along with an invitation to fill it out, along with a link. By signing an informed consent form, each respondent was fully informed about the study’s objectives and procedures prior to the questionnaire being distributed. Each participant filled out an anonymous questionnaire after separately giving their consent to participate, and they returned the questionnaire to the manager’s office in a designated box. Names and other identifying information of respondents were not collected. Data collection was performed from April to November 2022.

The research tool was a three-section questionnaire. The first section consisted of seven closed-ended questions (age group, sex, position held, department where he works, medical specialization), and two open-ended questions where the respondent had to fill in some details more fully, like the number of hours worked prior to and following COVID-19 and the number of weeks worked. The deeper exploration of the phenomena of violence served as the foundation for the remaining two sections. Four blocks of questions about workplace violence experiences were included in the first section, which asked about experiences before and during the COVID-19 pandemic. These questions asked for both quantitative and qualitative information about violence experiences, including details about the most significant violent incident that occurred at work. The final section asked questions about risk factors in place during their most serious event.

The study was approved by the Ethics Committee of “Nicolae Testemitanu” State University of Medicine and Pharmacy, Chisinau, the Republic of Moldova (Decision no. 2 from 24.01.2022).

### Data analysis

Data analysis was performed using statistical techniques appropriate to the data collected, including descriptive analysis. Frequency distributions (in percentage points) were calculated. To investigate whether there is a significant association between workplace violence phenomena before and during the COVID-19 pandemic, a series of analyses were conducted using the Pearson Chi-Square test, comparison by Wilcoxon signed rank test contrasting the replies of the respondents related to before and during COVID-19 periods and Wilcoxon signed rank test is the counterpart of Mann–Whitney test for the case of paired samples.

We employed binary logistic regression to explore the association between key demographic characteristics and various forms of workplace violence experienced by healthcare professionals. Three separate models were constructed, each with a distinct binary outcome variable: (1) having experienced serious workplace violence, (2) experiencing verbal threats during the COVID-19 pandemic, and (3) experiencing verbal threats prior to the pandemic. In all models, the outcomes were coded as 1 = Yes and 0 = No. The independent variables included in the analysis were sex (coded as 0 = Male [reference category], 1 = Female) and years of professional experience, categorized into three groups: 2–5 years (reference category), 6–10 years, and 11 years or more. These variables were selected based on their relevance to prior literature and the structure of our dataset.

Odds ratios (OR), 95% confidence intervals (CI), and corresponding *p*-values were reported for each predictor to assess the strength and significance of associations. Statistical significance was defined as *p* < 0.05.

For this, statistical calculation methods and formulas were applied using the IBM EXCEL program, IBM SPSS Statistics 27, with the help of the functions and modules of these programs. The data were organized into tables, analyzed, and quantitatively described.

## Results

### Demographic characteristics of respondents

Demographic characteristics varied significantly between professional groups (Table 1). The age distribution reveals that the majority of respondents were within the 40–49 age group, accounting for 31.7% of the sample. Gender distribution indicated a significant predominance of female participants, who made up 71.4% of the sample, compared to 28.6% male participants. Regarding professional roles, the largest group consisted of doctors, representing 43.9% of the participants, closely followed by nurses at 42.3%. In terms of professional experience, most participants (61.2%) had over 11 years of experience in healthcare services.

**Table 1:** Demographic distribution by categories and experiences of healthcare workers during the COVID-19 pandemic.

Characteristics	Nurses		Resident doctors		Doctors		Clinical support staff		ALL		Chi-square	*P*-value
	*N*	%	*N*	%	*N*	%	*N*	%	*N*	%		
Age											67.206	<0.0001
20–29	19	23.8	16	69.6	1	1.2	2	66.7	38	20.1		
30–39	15	18.8	7	30.4	24	28.9	0	0	46	24.3		
40–49	27	33.8	0	0	33	39.8	0	0	60	31.7		
50–59	16	20	0	0	19	22.9	1	33.3	36	19.1		
60+	3	3.8	0	0	6	7.2	0	0	9	4.8		
Total	80	100	23	100	83	100	3	100	189	100		
Sex											40.952	<0.0001
Male	4	5	11	47.8	39	47	0	0	54	28.6		
Female	76	95	12	52.2	44	53	3	100	135	71.4		
Total	80	100	23	100	83	100	3	100	189	100		
Years for working in the healthcare services											61.307	<0.0001
2–5	20	25.3	20	87	9	10.8	2	67	51	27.1		
6–10	5	6.3	3	13	14	16.9	0	0	22	11.7		
11 years or more	54	68.4	0	0	60	72.3	1	33	115	61.2		
Total	79	100	23	100	83	100	3	100	188	100		
Working in a COVID hospital/department at the time of data collection (April 2022-November 2022)											13.765	0.003
Yes	18	22.5	11	47.8	39	47	0	0	68	36		
No	62	77.5	12	52.2	44	53	3	100	121	64		
Total	80	100	23	100	83	100	3	100	189	100		
Main job department at the time of data collection												
Intensive care unit	15	19.0	3	13.0	13	15.9	0	0.0	31	16.6	50.555	0.243
Emergency room	19	24.1	7	30.4	14	17.1	0	0.0	40	21.4		
Postsurgical department	0	0.0	0	0.0	1	1.2	0	0.0	1	0.5		
Surgery	1	1.3	5	21.7	17	20.7	0	0.0	23	12.3		
Infectious disease department	29	36.7	1	4.3	22	26.8	1	33.3	53	28.3		
Internal medicine	4	5.1	2	8.7	2	2.4	0	0.0	8	4.3		
Obstetrics	8	10.1	0	0.0	1	1.2	0	0.0	9	4.8		
Other	3	3.8	5	21.7	12	14.6	2	66.7	22	11.8		
Total	79	19.0	23	13.0	82	15.9	3	0.0	187	16.6		

N, the absolute number; %, percent; Chi-square, Pearson’s chi-squared test; *P*-value, statistical significance indicator.

During the data collection period, 36% of the participants were working in a COVID-19 hospital or department. When examining the primary job departments, the highest percentage of participants worked in the infectious disease department (28.3%), followed by the emergency room (21.4%) and the intensive care unit (16.1%). Additional departments represented in the study include surgery (12.3%), obstetrics (4.8%), internal medicine (4.3%), and the postsurgical department (0.5%), with 11.8% of participants classified under “Other.”

### Clinical support staff

Clinical support staff (technical personnel), physicians, nurses, resident doctors, and other healthcare workers were divided into separate groups for the purpose of the study, which also assessed their demographics and experiences with workplace violence (Table 1). The data reveals significant differences across these groups in terms of age, gender, years of experience, and involvement in COVID-19 care.

There is a significant variation in age distribution among the different groups, with resident doctors predominantly in the 20–29 age group (69.6%), while doctors are more evenly distributed, with a notable proportion (39.8%) in the 40–49 age range. Gender differences are also prominent, with a higher proportion of females in the nursing category (95%) compared to other groups. The majority of nurses and doctors have over 11 years of experience (68.4 and 72.3%, respectively). Conversely, resident doctors predominantly have 2–5 years of experience (87%), reflecting their earlier stage in their professional careers. The involvement in COVID-19 care also varies significantly, with nearly half of the resident doctors (47.8%) and doctors (47%) working in COVID-19 departments during the data collection period. In contrast, a smaller proportion of nurses (22.5%) and none of the clinical support staff were involved in COVID-19 care. The significant chi-square values and *p*-values below 0.0001 for age, gender, years of experience, and involvement in COVID-19 care indicates that these differences are statistically significant.

### Experience of violent events before COVID-19 (March 2019 – March 2020)

Table 2 presents the pre-pandemic prevalence of workplace violence and associated worker perceptions. While physical assaults were comparatively rare, with 17.5% of respondents reporting such incidents, verbal threats or assaults affected 48.1% of the participants. Of the respondents, a minority reported sexual assault and harassment (2.7 and 2.1%, respectively). Stealing incidents were likewise comparatively rare, with 10.1% of participants reporting such incidents.

**Table 2:** Characteristics of experiencing a violent event.

Characteristics	Before COVID-19 (March 2019 – March 2020)		During COVID-19 (after January 2021)		*p*-values
	*N*	%	*N*	%	
Physical assault					
No	156	82.5	158	84.5	0,481*
Yes	33	17.5	29	15.5	
Total	189	100	187	100	
Verbal threat or assault					
No	98	51.9	104	55.3	0,248*
Yes	91	48.1	84	44.7	
Total	189	100	188	100	
Sexual assault					
No	183	97.2	182	97.8	1*
Yes	5	2.7	4	2.2	
Total	188	100	186	100	
Sexual harassment					
No	185	97.9	181	96.8	0,625*
Yes	4	2.1	6	3.2	
Total	189	100	187	100	
Theft					
No	170	89.9	167	89.8	1*
Yes	19	10.1	19	10.2	
Total	189	100	196	100	
Concerns regarding to workplace violence					
All the time	15	7.9	24	12.8	0,028**
Most of the time	22	11.6	21	11.2	
About half of the time	17	9	15	8	
Rarely	49	25.9	43	23	
Never	86	45.5	84	44.9	
Total	189	100	187	100	
Hospital prioritization of safety					
All the time	73	38.8	74	39.6	0,065**
Most of the time	44	23.4	30	16	
About half of the time	5	2.7	12	6.4	
Rarely	27	14.4	21	11.2	
Never	39	20.7	50	26.7	
Total	188	100	187	100	
Workplace violence affected work					
All the time	13	6.9	18	9.6	0,020**
Most of the time	17	9	16	8.6	
About half of the time	17	9	18	9.6	
Rarely	38	20.2	41	21.9	
Never	103	54.8	94	50.3	
Total	188	100	187	100	

N, the absolute number; %, percent; *, McNemar’s test; **, Wilcoxon paired signed-rank.

Concerns about workplace violence were expressed by 7.9% of respondents as being present all the time, and by 11.6% as being present most of the time. Though 45.5% never expressed concern, the majority did so either infrequently or never.

Medical institutions’ perceptions of safety prioritizing differed; 38.8% of respondents said their hospital prioritized safety all of the time, while 23.4% said it did so most of the time. On the other hand, 20.7% reported that safety was never prioritized.

Workplace violence frequently or mostly affected job performance for 15.9% of respondents. Even though workplace violence is a problem, not all workers will have the same level of impact on their ability to perform their jobs effectively, as demonstrated by the majority of respondents (54.8%) who said it has never harmed their work.

The McNemar’s test results had no statistically significant change in the frequency of specific types of workplace violence (e.g., physical assault, verbal threats, sexual assault, sexual harassment, or theft) when comparing the periods before and during the COVID-19 pandemic (Table 2). However, the Wilcoxon signed-rank test revealed significant shifts in participants’ concerns about workplace violence (*p* = 0.028) and perceptions of how violence affected their work (*p* = 0.020), with more respondents reporting persistent concerns and negative impact during the pandemic period. It is important to note that, in several cases, the data contained a high number of ties – participants reporting the same experience before and during the pandemic.

### Experience of violent events during COVID-19 (post – January 2021)

A detailed analysis of workplace violence experienced by healthcare staff during the COVID-19 pandemic (after January 2021), alongside their perceptions of safety and the impact of these incidents on their work was performed (Table 2).

The incidence of physical assault during the pandemic was slightly lower than in the pre-pandemic period, with 15.5% of respondents reporting such incidents. Verbal threats or assaults remained significant, affecting 44.7% of participants. Sexual assault was reported by 2.2% of respondents, while sexual harassment was experienced by 3.2%, a slight increase compared to the pre-pandemic data. Theft incidents were reported by 10.2% of participants, consistent with pre-pandemic levels.

Concerns about workplace violence increased during the pandemic, with 12.8% of respondents feeling concerned all the time, and 11.2% most of the time. Despite these concerns, nearly half of the respondents (44.9%) reported rarely or never being concerned about workplace violence.

The perception of safety prioritization within hospitals during the pandemic was mixed. While 39.6% of respondents believed that safety was prioritized all the time, this perception was lower than before the pandemic. Additionally, 26.7% of respondents reported that safety was never prioritized.

The impact of workplace violence on work performance during the pandemic was significant for some respondents, with 9.6% reporting that it affected their work all the time, and another 8.6% most of the time. However, 50.3% indicated that workplace violence never impacted their work, similar to pre-pandemic levels.

### Management and prevention of workplace violence

Table 3 presents data on the availability of training, policies, and institutional support related to workplace violence. The majority of participants (83.6%, *n* = 153) did not receive any training on workplace violence prevention, while only 16.4% (*n* = 30) reported having received such training. Among those who were trained, the topics covered included: the reasons behind violence against healthcare workers (14.5%, *n* = 9), methods to de-escalate situations that could lead to violence (33.9%, *n* = 21), hospital policies addressing workplace violence (30.6%, *n* = 19), and the resources available within the hospital to support victims of violence (21%, *n* = 13).

**Table 3:** Assessment of measures and perceptions regarding workplace violence prevention among healthcare professionals.

Characteristics	*N*	%
Have you received any training about workplace violence prevention		
No	153	83.6
Yes	30	16.4
Total	183	100
Topics included (multiple choice)		
Reasons for violence against healthcare workers	9	14.5
How to diffuse situations that could escalate to violence	21	33.9
Hospital policies that address workplace violence	19	30.6
Hospital resources to support you if you are a victim	13	21
Total	62	100
Does the leadership of the hospital consider violence prevention a priority		
No	76	41.8
Yes	106	58.2
Total	182	100
Does the hospital have an overall policy addressing workplace violence prevention		
No	70	38
Yes	114	62
Total	184	100
Does the hospital have a committee that addresses workplace violence		
No	121	66.5
Yes	61	33.5
Total	182	100
Does your hospital or unit have a security officer that you can call in case of danger		
No	93	50.8
Yes	90	49.2
Total	183	100
Existing workplace violence reporting system		
Yes	77	43.5
No	100	56.5
total	177	100
Existence of action taken by the hospital to reduce workplace violence		
Yes	51	28.8
No	126	71.2
Total	177	100

N, the absolute number; %, percent.

Leadership commitment to violence prevention is perceived as a priority by 58.2% of respondents, while 41.8% believe that their hospital leadership does not prioritize this issue. Despite this, 62% of respondents acknowledged the existence of an overall policy addressing workplace violence prevention in their hospitals. However, only 33.5% reported the presence of a dedicated committee to address workplace violence. The availability of security officers is nearly split, with 50.8% of respondents reporting the absence of security personnel they can call in case of danger. Additionally, only 43.5% of respondents reported the existence of a workplace violence reporting system, while a concerning 56.5% stated that no such system is in place.

Furthermore, 71.2% of respondents indicated that no actions had been taken by their hospital to reduce workplace violence.

Environmental factors contributing to workplace violence were also noted, with 38.5% (*n* = 82) identifying areas where they could become isolated or cornered, 34.3% (*n* = 73) reporting poor visibility areas, and 27.2% (*n* = 58) pointing to areas where patients are not easily monitored by staff. It appears from the data that some aspects of the surroundings in healthcare institutions are thought to raise the possibility of violence at work. These elements, which include poorly lit rooms, places where isolation is possible, and areas with insufficient patient supervision, might produce circumstances that make healthcare personnel feel exposed.

### The nature, severity, and range of responses to acts of violence against medical staff

Nearly half of the healthcare professionals (43.1%) have encountered severe violence in their work environment. Of these, 60.8% were verbal threats or assaults; 29.1% involved physical assault. Sexual assault and harassment were each reported by 2.5% of respondents as the most serious events experienced.

Most violent incidents were perpetrated by patients (45.6%) and their family members/acquaintances (39.2%), 6.3% of incidents involved coworkers, and 5.1% involved managers or supervisors.

Violent incidents were almost equally distributed between day shifts (51.3%) and night shifts (48.7%). The majority of incidents (79.5%) occurred during weekdays, with only 20.5% happening on weekends (Table 4).

**Table 4:** Characteristics of the nature and severity of violence cases.

Characteristics	*N*	%
Experience of a very serious event at any time in the career		
Yes	81	43.1
No	107	56.9
Total	188	100
Most serious event		
Physical assault	23	29.1
Verbal threat or assault	48	60.8
Sexual assault	2	2.5
Sexual harassment	2	2.5
Theft	4	5
Total	79	100
Perpetrator		
Patient	36	45.6
A patient’s family member/acquaintance	31	39.2
Coworker	5	6.3
Manager/supervisor	4	5.1
Other	3	3.8
Total	79	100
Time of occurrence I		
Day shift	40	51.3
Night shift	38	48.7
Total	78	100
Time of occurrence II		
During weekdays	62	79.5
During weekends	16	20.5
Total	78	100

N, the absolute number; %, percent.

### Workplace violence before and during the pandemic

The relationships between workplace violence experiences before the pandemic (March 2019 – March 2020) and during the COVID-19 pandemic (post – January 2021) for various incident types reveal important insights into the prevalence of various forms of workplace aggression and misconduct. The paired samples test did not find a significant change in the overall frequency of physical assaults (*p* = 0.347), the slight increase is not statistically significant at the group level.

For verbal threats, the statistics indicate a marginal increase in the average of verbal threats or assaults. The correlation is very strong (0.714), meaning a high level of consistency in individual experiences. The paired samples test underlines that this increase is not statistically significant (*p* = 0.179), meaning that the observed increase does not represent a significant change at the group level.

For sexual assaults, the average frequency remained unchanged. The correlation is moderate (0.434). The paired samples test confirms the absence of a significant change (*p* = 1.000) – the frequency of sexual assaults did not change before and during the COVID-19 pandemic.

Statistics reveal a negligible decrease in the average of sexual harassment, with a strong correlation (0.602). The paired samples test did not find a significant change (*p* = 0.319), this decrease was not statistically significant at the group level.

In the case of thefts, the statistics demonstrate that there is no change in the average number of theft incidents. The correlation is moderate (0.472). The paired samples test confirms the absence of a significant change (*p* = 1.000) – the frequency of thefts remained stable before and during the pandemic.

Logistic regression analysis (Table 5) was conducted to explore demographic predictors of workplace violence. Female healthcare workers had significantly lower odds of reporting serious violence during their careers compared to males (OR = 0.43, 95% CI: 0.22–0.84, *p* = 0.013). Experience level was also a significant factor: participants with 11 or more years of experience had consistently lower odds of reporting violence across all models. In contrast, those with only 2–5 years of experience were at higher risk. The subgroup with 6–10 years of experience has inconsistent results with wide confidence intervals.

**Table 5:** Logistic regression analysis of demographic predictors of workplace violence experiences.

Dependent variable	Experience of serious violence in career		Experiencing verbal threat during COVID-19		Experiencing verbal threat before COVID-19	
	OR (95% CI)	*p*-value	OR (95% CI)	*p*-value	OR (95% CI)	*p*-value
Sex						
Males	Ref.	–	Ref.	–	Ref.	–
Females	0.43 (0.22–0.84)	0.013	0.65 (0.33–1.27)	0.207	0.65 (0.33–1.28)	0. 217
Years of experience						
2–5 years	Ref.	–	Ref.	–	Ref.	–
6–10 years	0.69 (0.24–1.95)	0.482	2.64 (0.84–8.30)	0.097	1.89 (0.60–6.0)	0.280
11 years and more	0.30 (0.14–0.57)	<0.001	0.39 (0.20–0.78)	0.007	0.31 (0.16–0.62)	<0.001

## Discussion

Our findings emphasize the importance of understanding healthcare workers’ experiences during the COVID-19 pandemic. This is especially relevant for middle-aged professionals in high-stress, critical care settings such as infectious disease units, emergency rooms, and intensive care units. Our findings indicate that although the reported prevalence of workplace violence events did not increase from prior to during the pandemic, concern about violence increased. Healthcare practice during the pandemic, which restricted the number of visitors in the hospital and isolated patients, may have contributed to decreased risk. However, the stressful environment of the COVID-19 pandemic treatment period may have exacerbated violence risk and perception. A recent scoping review (16) of qualitative research on healthcare workers’ experiences during COVID-19 highlights the importance of this assessment, as by identifying key challenges, strengths, and intervention points, the review provides valuable insights for strategies aimed at strengthening healthcare workers and improving their experiences. Violence against healthcare workers is a critical issue that affects the quality of healthcare services and the safety of both healthcare workers and patients (17).

In our study, before the COVID-19 pandemic, workplace violence in healthcare primarily involved verbal threats, affecting nearly half of the workforce (48.1%), physical assaults were less frequent (17.5%), and sexual harassment was rare. However, during the pandemic, while physical assaults slightly decreased (15.5%), sexual harassment (3.2%), and concerns about workplace violence intensified (from 45.5 to 50.3%), reflecting a shift in the nature and impact of these incidents. Strong correlations (e.g., *r* = 0.654 for physical assault) suggest that individual experiences with workplace violence remained consistent over time. In most categories, these correlations indicate stable exposure patterns, with no statistically significant group-level changes. Zhang et al. (18) identified an increase in workplace violence among medical staff during the pandemic, with verbal violence affecting 48%, physical violence 9%, and emotional violence 26% of healthcare workers; incidents rose from mid to late pandemic, with physical violence increasing from 12 to 23%. Also, the authors mentioned in their results that nurse faced more than twice the rate of physical violence compared to physicians, no correlation was found between workplace violence and factors like gender, profession, or pandemic timing. Contrarily, our study revealed significant demographic and professional differences among healthcare worker groups. Resident doctors were generally younger and less experienced, while doctors and nurses were older and had more years of service. Gender disparities were also evident, particularly within nursing roles. Regression findings support our observation that less experienced healthcare workers, such as resident doctors, may be more vulnerable to workplace violence. The data underline a clear inverse association between years of experience and reported violence exposure. While female workers reported lower odds of serious violence, gender differences were not statistically significant for verbal threats specifically during or before COVID-19. The data on how healthcare workers responded to violent incidents reveals a diverse range of actions, reflecting both immediate reactions and longer-term coping strategies.

The most common response, reported by 40.3% of participants, was to directly confront the perpetrator by telling them to stop the violent behavior. In addition to verbal confrontation, 13.4% of respondents turned to their colleagues for support. Similarly, 11.2% shared their experiences with family or friends. Interestingly, 11.9% of healthcare workers attempted to defend themselves physically, and the same percentage chose to report the incident to a senior staff member. The willingness to engage in physical self-defense underscores the real and perceived physical risks involved, while reporting to senior staff indicates that formal reporting mechanisms are utilized, albeit by a relatively small proportion of workers. However, not all healthcare workers felt empowered to act. A small but significant 5.2% of respondents reported taking no action in response to the violence they experienced. This lack of response could point to a sense of helplessness, fear of retaliation, or possibly a workplace culture where violence is insufficiently addressed or even normalized.

Further, 3.7% of participants opted to transfer to another position following a violent incident, reflecting the severity of their experiences and the impact on their career choices. The least common response was contacting emergency services, reported by just 2.2% of workers.

Our findings reveal critical gaps in workplace violence preparedness at the institutional level. Most healthcare workers had not received formal training, and many reported the absence of both reporting systems and on-site security presence. These conditions may not only increase vulnerability but also reduce trust in institutional support, discouraging workers from reporting incidents when they occur. Several studies identified significant gaps in workplace violence prevention within healthcare settings, similar to our findings. For instance, a report by the American Hospital Association (19) emphasizes that many healthcare institutions lack comprehensive training programs, security personnel, and effective reporting systems, which are crucial for mitigating workplace violence. It also notes that only a fraction of hospitals has dedicated committees or proactive measures to address violence. In 2021, the Joint Commission’s R3 Report provides detailed rationale and references for developing new requirements to improve healthcare standards (20).

A considerable portion of healthcare workers reported a lack of workplace violence training, no access to security officers, and the absence of formal reporting mechanisms, pointing to structural gaps that leave staff vulnerable to violent incidents, particularly in high-stress environments such as COVID-19 units. Improving facilities like lighting, isolation reduction, and staffing are crucial, being part of broader strategies to address the root causes of workplace violence and protect healthcare professionals from harm (18). These findings align closely with the recent study, reinforcing the need for healthcare facilities to prioritize environmental improvements as part of comprehensive workplace violence prevention programs.

According to our results, nearly half of healthcare professionals have experienced severe violence in their work environment. Verbal threats and assaults are the most common. Most violent incidents are perpetrated by patients and their families, with a smaller percentage involving coworkers and managers. Violence occurs equally across day and night shifts, primarily during weekdays. Recent studies on workplace violence in healthcare settings (21) – research indicates that violence in healthcare settings is a significant and growing problem, with nearly 75% of the approximately 25,000 workplace assaults are reported annually in these environments. Brito and Hasselmann (21) mentioned that security measures in healthcare facilities vary widely, from lacking formal security forces to employing dedicated departments or contracting out services. According to our results, healthcare workers responded to violent incidents in various ways – most often by confronting the perpetrator, seeking support, or reporting to senior staff. Some chose not to act, possibly due to workplace culture or feeling powerless, while a few even changed jobs. Emergency services were rarely contacted.

The findings of this study must be interpreted in the context of broader regional patterns. While the current results focus on healthcare workers in Moldova, this study is part of a larger international project conducted across Romania, Georgia, and Armenia. Comparative results from the four countries will be published separately and will provide a more detailed regional analysis. Although this study focuses on six hospitals in the Republic of Moldova, the findings reflect broader challenges observed in many low- and middle-income healthcare systems. Issues such as insufficient workplace violence training, lack of reporting systems, and environmental safety risks are not unique to Moldova and have been reported in other Eastern European and post-Soviet countries. Therefore, the results may be generalized to similar contexts where healthcare systems face resource constraints and evolving occupational safety standards.

In the Republic of Moldova, according to the latest amendments to the Penal Code (22) and the Contravention Code (23), violence against doctors and medical workers during service will be sanctioned. According to these two documents, premeditated offenses against the honor, dignity, or professional reputation of a medical worker will incur a fine ranging from 500 to 1,250 lei. Insulting a medical worker, when accompanied by hooligan actions and bodily harm, will result in a fine of up to 7,500 lei or a contravention arrest of up to 15 days. Deliberate actions that undermine the honor, dignity, or professional reputation of doctors or medical personnel while performing their duties will incur a penalty of up to 1,250 lei, imposed on the individual responsible. The outrage, accompanied by acts of hooliganism and/or bodily harm, will be sanctioned with fines of up to 7,500 lei, applied to the natural person or with contravention arrest of up to 15 days.

Our findings bring attention to urgent need for concrete measures to prevent and manage workplace violence in healthcare institutions in the Republic of Moldova. The lack of formal training, reporting mechanisms, and security personnel in high-risk departments underscores the vulnerability of healthcare workers and the necessity for institutional intervention. Implementing regular training programs focused on recognizing, preventing, and reporting violence, alongside the development of confidential and efficient reporting systems, is essential. At the same time, hospitals must ensure a visible security presence and invest in safety infrastructure. The results also emphasize the importance of providing psychological support for affected staff and fostering an institutional climate that encourages response and reporting. In addition, internal protocols must be aligned with recent legislative changes to ensure that penalties for violence against healthcare workers are effectively enforced. This study also supports the need for a systematic national monitoring approach, enabling the development of coherent protective policies and the appropriate allocation of resources for healthcare worker safety.

In addition, this study can serve as a foundation for future research, including comparative regional analyses, intervention-based studies, and longitudinal research on workplace violence prevention. The data may also inform national health policy discussions and guide institutional improvements in both Moldova and other countries with similar healthcare infrastructures.

### Limitations

This study has several limitations that should be considered when interpreting the findings. First, the data rely on self-reported experiences, which are subject to recall bias, particularly for events reported from the pre-pandemic period (March 2019 – March 2020). Although time anchors were provided in the questionnaire to improve accuracy, retrospective data may still be imprecise. There is also a possibility of social desirability bias, where respondents might underreport incidents of violence or overreport institutional responses due to perceived expectations.

Second, the study is subject to selection bias, as participation was voluntary – it is possible that individuals with more exposure to or stronger opinions about workplace violence were more likely to respond and may have attracted individuals who had stronger opinions or direct experiences with workplace violence. The 36% response rate and estimated 18% non-response rate may also introduce non-response bias, as the views of non-participants could differ from those who responded.

Third, the cross-sectional design limits the ability to establish causal relationships between risk factors and workplace violence or to assess true trends over time. While we compared experiences before and during the pandemic, these snapshots do not reflect longitudinal changes.

The direction of potential bias may include both underestimation (due to stigma or fear of disclosure) and overestimation (if individuals with more frequent or severe experiences were more motivated to respond). Although we attempted to minimize bias through anonymous responses and standardized instructions, pointing to for longitudinal and multi-center studies in future research.

Future studies using longitudinal or prospective designs could provide more robust comparisons.

## Conclusion

This study is the first systematic, cross-sectional assessment of workplace violence experienced by healthcare professionals in the Republic of Moldova before and during the COVID-19 pandemic. Although the overall frequency of workplace violence, including physical assault, verbal threats, sexual harassment, and theft did not significantly change during the pandemic, concerns about workplace violence and its perceived impact on professional performance increased. Verbal threats remained the most frequently reported form of violence in both periods, while physical assaults – a slight but statistically non-significant decrease. Healthcare workers with more than 11 years of experience were significantly less likely to report serious incidents of violence, while those with only 2–5 years of experience were at elevated risk. Female healthcare workers had significantly lower odds of reporting serious workplace violence compared to males; however, gender differences were not significant for verbal threats specifically during or before the pandemic. In addition to individual vulnerability, institutional and environmental risk factors were identified.

These findings indicate that workplace violence remains a pervasive issue across all shifts and departments, disproportionately affecting younger and less experienced staff. Addressing these challenges requires targeted institutional measures, including training, improved physical infrastructure, reporting systems, and ongoing monitoring to reduce risks and support frontline healthcare workers effectively.

## Data Availability

The raw data supporting the conclusions of this article will be made available by the authors, without undue reservation.
